# The epidemiology of upper respiratory tract disorders in a population of insured Swedish dogs (2011–2014), and its association to brachycephaly

**DOI:** 10.1038/s41598-023-35466-0

**Published:** 2023-05-30

**Authors:** M. Dimopoulou, K. Engdahl, J. Ladlow, G. Andersson, Å. Hedhammar, E. Skiöldebrand, I. Ljungvall

**Affiliations:** 1 Department of Clinical Sciences, Swedish University of Agricultural Sciences, Uppsala, Sweden; 2 Department of Veterinary Medicine, University of Cambridge, Cambridge, UK; 3 Department of Animal Breeding and Genetics, Swedish University of Agricultural Sciences, Uppsala, Sweden; 4 Department of Biomedical Science and Veterinary Public Health, Swedish University of Agricultural Sciences, Uppsala, Sweden

**Keywords:** Respiratory tract diseases, Epidemiology

## Abstract

Upper respiratory tract (URT) disorders are common in dogs but neither general nor breed-related epidemiological data are widely reported. This study´s aims were to describe the epidemiology of URT disorders in a Swedish population of dogs and to investigate whether brachycephalic breeds were overrepresented among high-risk breeds. A cohort of dogs insured by Agria Djurförsäkring in Sweden (2011–2014) was used to calculate overall and breed-specific incidence rate (IR), age at first URT diagnosis and relative risk (RR) for URT disorders. For breeds with high RR for URT disorders, co-morbidities throughout the dog’s insurance period and age at death were investigated. The cohort included approximately 450,000 dogs. URT disorders had an overall IR of 50.56 (95% CI; 49.14–52.01) per 10,000 dog years at risk. Among 327 breeds, the English bulldog, Japanese chin, Pomeranian, Norwich terrier and pug had highest RR of URT disorders. Eight of 13 breeds with high RR for URT disorders were brachycephalic. The median age at first URT diagnosis was 6.00 years (interquartile range 2.59–9.78). French bulldogs with URT diagnoses had a significantly shorter life span (median = 3.61 years) than other breeds with URT diagnosis (median = 7.81 years). Dogs with high risk for URT disorders had more co-morbidities than average.

## Introduction

Upper respiratory tract (URT) disorders comprise a group of diseases with heterogeneous morbidity, ranging from mild transient disorders like virus infections, to potentially life-threatening disorders like brachycephalic obstructive airway syndrome (BOAS). Although URT disorders are considered common in veterinary practice^1–3^, large-scale epidemiological studies investigating the overall and breed-specific occurrence of URT disorders in dogs are currently scarce in the veterinary literature. Canine brachycephaly has often been correlated with URT disorders^4–8^. A recently published study from the UK, which was based on primary care clinical data from over 90 clinics, investigated the prevalence of URT disorders in three brachycephalic and three non-brachycephalic dog breeds^4^. The authors reported that URT disorders were common, and that the brachycephalic dogs had 3.5 times the odds of being diagnosed with an URT disorder compared to the three non-brachycephalic breeds investigated^4^. However, hospital populations may not be representative of the population at risk, introducing selection bias when disease prevalence is estimated.

Although several case series describe breed-specific disorders, such as BOAS in the French and English bulldog and the pug^7,9^, sleep-disordered breathing in the Cavalier King Charles Spaniel (CKCS)^10^,upper airway obstruction in the Norwich terrier^11^ and pneumonia in the Irish wolfhound^12^ data on breed-specific incidence and risk for URT disorders are scarce. Breed-specific estimates of disease occurrence are important for increasing awareness of breed susceptibilities to health problems among veterinarians, animal owners, breeders and researchers. Such data can consequently help in diagnostic and treatment processes, health-promoting breeding strategies, and form a basis for further research.

Estimates of disease incidence and prevalence that can be generalised to the entire population at risk are necessary in order to evaluate the impact of URT disorders at a population level. Large-scale epidemiological data can be retrieved from insurance databases, which provide demographic and diagnostic information on both clinical cases and the healthy, insured background population, Agria Djurförsäkring is the largest insurance company for pets in Sweden, and covered approximately 50% of the insured dog population in 2012^13^ In Sweden, the vast majority of dogs (77%) were insured in 2012^14^, rending the Agria-insured dog population representative of the national dog population^15,16^. Canine epidemiological studies based on data from Agria Djurförsäkring have been published for various conditions such as cruciate ligament rupture, chronic kidney disease, diabetes mellitus, dystocia, and atopic dermatitis,^13,17–20^ but not for respiratory disorders. The historical insurance data from the Agria database (2011–2014) are of especially high value for investigating the epidemiology of URT disorders, because after this period, an exemption for all veterinary care events involving upper airway disease was introduced for Boston terriers, English and French bulldogs, and pugs. This means that the database after 2014 contains no new registrations of upper airway conditions in these breeds.

Most epidemiological studies report general and breed-specific frequency of the disorder of interest. Analysis of co-morbidity by investigating concurrent diagnoses in the breeds of interest provides more information on the general health status of these breeds. This can be of use for clinical and health-promoting practices, and lead to further research on possible disorder correlations.

The primary aims of this study were to provide population-based estimates of the incidence of URT disorders, to calculate relative risk (RR) stratified by breed and sex and to investigate if brachycephalic breeds were among the top breeds-at-risk for URT disorders in a cohort of insured Swedish dogs. Additional aims were to report the age at first URT diagnosis and age at death for dogs with URT diagnosis in high-risk breeds and to investigate whether dogs at high risk of URT disorders had increased co-morbidity throughout their insurance period compared to the general dog population.

## Methods

This was a retrospective single cohort study based on data from Agria Djurförsäkring in Sweden. The insurance process has previously been described in detail^21^. There were two types of insurance: veterinary care and life, and dogs could be insured in either one or both. For dogs with veterinary care insurance, veterinary care costs were reimbursed and the insurance claim registered in the database if the costs exceeded the deductible amount. The veterinary care insurance had no upper age limit, while life insurance was terminated at the age of eight, 10, or 12 years, depending on breed (see supplementary Table 1), and owners were reimbursed when the dog died or was euthanised. Claims for conditions not covered by the insurance (for example vaccination, dental care, and certain congenital disorders) were not registered. For URT disorders, the only exemptions from veterinary care insurance during this period were for the surgical treatment of conditions affecting the nostrils, soft palate, trachea and pharynx in the Boston terrier, French and English bulldog and pug. However, owners of the aforementioned breeds were reimbursed for diagnosis and medical treatment of conditions affecting the upper airways throughout the observation period. After April 1^st^, 2014, the Boston terrier, French and English bulldog and the pug were excluded from all veterinary care for conditions affecting the nostrils, soft palate, trachea and pharynx.

### Study population

Inclusion criteria were all dogs insured for veterinary care by Agria Djurförsäkring, Sweden at any time between 1^st^ January 2011 and 31^st^ March 2014. Sex was recorded as either female or male, as information on neutering status was unavailable. Exclusion criteria were dogs solely enrolled for life insurance and dogs for which information about breed, sex, birth year, age or date of enrolment was uncertain or missing.

### Data collection

For each dog the following variables were retrieved from the database: date of birth, date of death (if available), breed, sex, date when the dog entered and left the insurance programme, type of insurance (veterinary care or veterinary care and concurrent life insurance), date of claim and diagnostic code(s) (if any).

### Diagnostic classification

The diagnostic code(s) were assigned by the attending veterinarian based on the hierarchical diagnostic registry developed by the Swedish Association of Veterinary Clinics and Hospitals^22^. Diagnoses in this registry are classified according to organ system, for example cardiovascular or respiratory, and disease process, for example neoplastic or inflammatory. The date of diagnosis and death in the Agria database corresponded to the date when the claim was registered. If receipts from several veterinary appointments within a 125-day insurance period were submitted at the same time, these were usually coded in separate claims but registered at the same date. For the purposes of this study the upper respiratory system included the trachea, but not the bronchi^23,24^. The URT diagnostic codes used included both presenting complaints such as “cough from the upper airway”, “increased noise from the upper airway” and more specific diagnoses, such as “elongated soft palate”, “tracheal collapse”, and are listed in supplementary Table 2.

### Data analysis

Data analysis was performed in RStudio version 1.2.1335^25^. Summary statistics for continuous variables are presented as median (Interquartile range, IQR) and for categorical variables as number (percentage). Incidence rate (IR) is expressed as the number of dogs with URT claims per 10,000 dog-years-at-risk (DYAR). Only the first URT claim for each dog was used for IR calculation. Dogs with no URT claims contributed to the total DYAR with the time they were insured within the observation period, and dogs with URT claims contributed from the start of the observation period until the time of the first URT claim. Relative risk (RR) was calculated by dividing the IR of the subgroup of interest (e.g. breed or sex) with the IR of the rest of the population. The 95% confidence intervals (95% CI) for IR and RR were calculated with the R-package “exactci” (version 1.3–3), based on the Poisson distribution^26^. Bonferroni (BF) correction for multiple comparisons was used to adjust the significance level and p-values of < 0.05 after corrections were considered significant. A forest plot from the R-package “forestplot” (version 1.9) was used to describe breed risks^27^.

Breeds with significantly higher RR for URT diagnosis were termed “high-risk breeds”. Median age at first URT diagnosis was calculated for high-risk breeds, and Wilcoxon rank sum test was used to compare the age at first URT-diagnosis between breeds, and between brachycephalic and non-brachycephalic breeds. In dogs with URT diagnoses and concurrent life insurance, age at life claim (age at death) was calculated for dogs that died/were euthanised during the observation period. The age at death for each high-risk breed was compared to the age at death for all other breeds with URT diagnoses. Only high-risk breeds with more than six cases of death/euthanasia were included in this analysis.

Co-morbidity was evaluated for dogs of high-risk breeds. For dogs with URT claims, previous and subsequent diagnoses from the start of the observation period until 31 December 2016 or withdrawal from the insurance were retrieved. Each high-risk dog was compared with five randomly chosen age-matched control dogs (= dogs with no URT diagnosis). Previous/subsequent diagnoses were compared between cases and controls using conditional logistic regression, with URT-diagnosis as the main exposure variable and previous/subsequent diagnoses as outcome. For cases, co-morbidities diagnosed at the same date as the first URT diagnosis were excluded.

## Results

### Study population

During the period 1 January 2011 to 31 March 2014, there were 453,548 dogs with veterinary care insurance and complete demographic data in the database, contributing 951,136 DYAR. Each dog contributed with a median of 2.33 years (IQR 0.99–3.25). Descriptive features of the study population are presented in Table 1.

**Table 1:** Descriptive features of dogs with veterinary care insurance in Agria Djurförsäkring in Sweden during the period 1 January 2011 to 31 March 2014.

Number of dogs with veterinary care insurance	453,548
Number of dogs with concurrent life insurance	291,319
Total duration of insurance	951,136 y
Insurance duration in years, median (IQR)*	2.33 y (0.99 y—3.25y)
Age at observation start, median (IQR)	3.04 y (0.59 y—6.22y)
Age at insurance enrolment, median (IQR)**	16.0 w (9 w–2.60 y)
*Sex (%)*	
Female	223,032 (49.2%)
Male	230,516 (50.8%)

*IQR* interquartile range, *y* years, *w* weeks.

*Per dog, during the observation period.

**For dogs insured after 1 January 2011.

**Figure 1:**
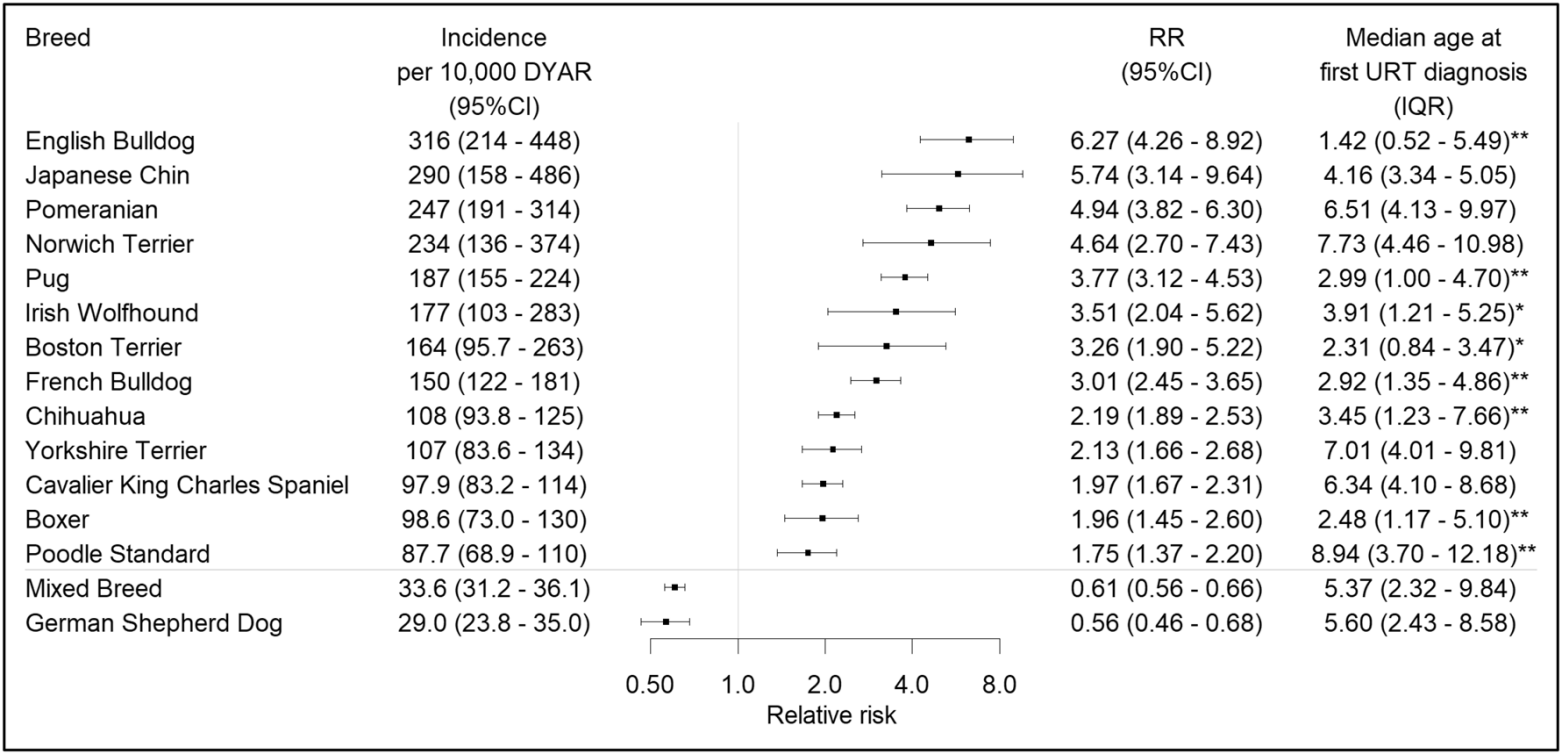
Breeds with significantly higher and lower RR for URT disorders after BF correction (significance level after BF correction 0.05/327). Breeds above the grey line had a significantly increased RR, and mixed breed and German shepherd dog (below the grey line) had a significantly decreased risk. *age at first URT diagnosis significantly different from all 205 breeds with URT diagnosis. **age at first URT diagnosis significantly different from 205 breeds with URT diagnosis after BF correction (significance level after BF correction 0.05/205). *DYAR*: dog years at risk; *CI:* confidence interval; *RR*: relative risk; *URT:* upper respiratory tract; *BF*: Bonferroni.

A total of 6590 claims for URT disorders were registered in 4781 dogs. The overall IR for URT disease was 50.56 (CI; 49.14–52.01) cases per 10,000 DYAR. Of 327 breeds in the database, 205 breeds had at least one dog with an URT veterinary care diagnosis. Thirteen breeds had a significantly increased risk and two breeds a significantly decreased risk for URT disorders after BF correction (Fig. 1). Among 13 high-risk breeds, eight were brachycephalic and five were non-brachycephalic (Table 2). The English bulldog, Japanese chin, Pomeranian, Norwich terrier, and pug had the highest IR of URT disorders. Median age at first URT diagnosis was 6.00 years (IQR 2.59–9.78), and varied with breed. The English and French bulldog, pug, Chihuahua and Boxer had a significantly lower age than average at first URT diagnosis after BF correction (Fig. 1). Brachycephalic high-risk breeds were younger (*p* < 0.001) at first URT diagnosis (4.29 years, IQR; 1.73–6.66) compared to non-brachycephalic high-risk breeds (4.97 years, IQR; 1.83–9.19).

**Table 2:** Brachycephalic and non-brachycephalic high-risk breeds with URT diagnoses in a cohort of insured Swedish dogs.

Brachycephalic breeds	Non-Brachycephalic breeds
Boxer	Chihuahua
Boston terrier	Irish Wolfhound
CKCS	Norwich terrier
English bulldog	Poodle standard
French bulldog	Yorkshire terrier
Japanese Chin	
Pomeranian	
Pug	

Male dogs had an increased risk of having an URT diagnosis compared to female dogs (RR 1.19, CI; 1.12–1.26, *p* < 0.001). The pug was the only high-risk breed for which male dogs had an increased risk compared to female dogs, after BF correction (RR 1.91, CI; 1.28–2.89, *p* < 0,001). Of 935 dogs of a high-risk breed with an URT diagnosis in the veterinary care database, 682 (72.9%) had a concurrent life insurance. Of these, 140 dogs (20.5%) had a life insurance settlement, irrespective of cause, during the observation period. The median age at life claim event (death or euthanasia) in all dogs with URT-diagnoses was 7.81 (IQR; 5.66–9.31) years and varied with breed (Table 3). French bulldogs with URT diagnosis were significantly younger (3.62 years, IQR; 1.89–6.72) at death/euthanasia compared to all other dogs with URT diagnosis, after BF correction.

**Table 3:** Median age at death/euthanasia for high-risk breed dogs with URT diagnoses in a cohort of insured Swedish dogs. Only breeds with more than six dogs are included.

Breed	Median age at life claim event (years, IQR, range)	Number of dogs
Boxer	7.22 (6.12–7.82, 1.12–10.11)	10
CKCS	8.63 (7.70–9.28, 1.24–9.92)	29
Chihuahua	8.13 (6.31–9.63, 0.80–11.96)	20
English bulldog	6.71 (5.93–8.98, 0.24–10.04)	6
French bulldog	3.62* (1.89–6.72, 0.81–8.55)	12
Irish wolfhound	4.93 (2.59–6.15, 0.81–7.56)	7
Pomeranian	8.29 (5.90–9.23, 2.61–10.08)	12
Standard poodle	8.48 (6.75–9.39, 4.82–10.06)	7
Pug	5.90 (4.70–8.07, 2.10–9.47)	15
Yorkshire terrier	8.68 (7.70–9.16, 3.36–9.92)	9

*The French bulldog was the only breed with significantly lower median age at death/euthanasia after BF correction, compared to all other dogs with URT-diagnoses (significance level after correction 0.05/13).

The odds of having registered co-morbidities before/after the first URT claim, compared to age-matched controls, varied with breed and is detailed in Table 4. With the exception of the Boston-, Norwich-, Yorkshire terrier, and Japanese chin, all other high-risk breeds had increased odds for co-morbidity during the observation period. The boxer, CKCS, Chihuahua, French bulldog, pug and standard poodle had high risk for co-morbidity both prior and after the first URT claim.

**Table 4:** Odds ratios for previous and subsequent diagnoses for 13 breeds with increased RR for URT disorders, compared to age-matched controls, in a Swedish cohort of insured dogs.

Breed	OR of prior diagnosis to first URT claim, with 95% CI	OR of subsequent diagnosis to first URT claim, with 95% CI
**Boston terrier**	7.41 (1.92–28.6, *p* = 0.003)	25.41 (3.20–201.60,* p* = 0.002)
**Boxer**	7.60 (3.81–15.17, *p* < 0.001)*	9.83 (4.00–24.18, *p* < 0.001)*
**CKCS**	3.24 (2.26–4.65, *p* < 0.001)*	6.40 (4.02–10.21, *p* < 0.001)*
Chihuahua	2.48 (1.78–3.46, *p* < 0.001)*	4.34 (3.01–6.25, *p* < 0.001)*
**English bulldog**	3.79 (1.66–8.63,* p* = 0.001)*	3.99 (1.65–9.68,* p* = 0.002)
**French bulldog**	4.01 (2.56–6.27, *p* < 0.001)*	4.51 (2.79–7.30, *p* < 0.001)*
Irish wolfhound	7.11 (2.19–23.11,* p* = 0.001)*	2.80 (0.91–8.63,* p* = 0.072)
**Japanese chin**	0.70 (0.19–2.65,* p* = 0.602)	4.29 (1.06–17.31,* p* = 0.040)
Norwich terrier	1.65 (0.56–4.86,* p* = 0.364)	1.66 (0.54–5.06,* p* = 0.373)
**Pomeranian**	2.26 (1.31–3.91,* p* = 0.003)	3.21 (1.80–5.73, *p* < 0.001)*
**Pug**	4.06 (2.60–6.33, *p* < 0.001)*	5.13 (3.20–8.24, *p* < 0.001)*
Standard poodle	4.38 (2.51–7.66, *p* < 0.001)*	6.72 (3.56–12.69, *p* < 0.001)*
Yorkshire terrier	1.75 (1.05–2.91, *p* = 0.030)	2.03 (1.21–3.41, *p* = 0.008)

Breeds in bold text = brachycephalic breeds.

*OR;* odds ratio.

*OR significantly increased after BF correction (significance level after BF correction 0.05/26).

## Discussion

To our knowledge, this is the first published large-scale study to report the epidemiology of URT disorders with focus on breed-specific risks. We found that URT disorders were common, and identified 13 high-risk breeds, eight of which were brachycephalic. High-risk dog breeds had their first URT diagnosis at a median age of approximately 6 years, and had an increased disease burden compared to average. Brachycephalic high-risk breeds acquired their first URT diagnosis at a younger age than non-brachycephalic high-risk breeds.

The overall IR of URT diagnosis was 50.56 cases per 10,000 DYAR in the cohort studied. This is comparable to the incidence of respiratory disorders reported in a recent study on presenting complaints and diagnosis distribution in Australian dogs based on insurance data^3^. Respiratory disorders were the fifth most common diagnostic category after gastrointestinal, dermatological, urogenital and other (unspecified) disorders in an earlier study on the distribution of diagnosis in Swedish dogs insured in Agria Djurförsäkring^1^.

Thirteen breeds were identified with increased risk of URT disorders. Two of these, the Japanese chin and standard poodle, which are relatively uncommon breeds, have to our knowledge no previously reported URT disorder predisposition. It is possible that our results reflect a regional disease variation, or, that URT disorders not historically common in these breeds are becoming increasingly prevalent, or, that being uncommon breeds, predispositions may be difficult to identify. The Japanese chin, a brachycephalic breed, has recently been included in case series on BOAS and epiglottic retroversion (displacement of the epiglottis into the larynx lumen resulting in inspiratory airflow limitation and/or distress)^28,29^ and being a toy breed, may have a predisposition to tracheal collapse^30,31^. Toy breeds like the Japanese chin, Chihuahua and Pomeranian have undergone exaggerated miniaturization. As a consequence, the vomerine alae can be too large in relation to the lumen, leaving an air passage of 1 mm or less at the level of the meatus nasopharyngeus (the passage from the nasal cavity to the pharynx)^32^. This can result in stertorous breathing, excessive reverse sneezing and nasal discharge, which are common URT presenting complaints in these breeds^32^.

All remaining 11 high-risk breeds have a previously reported susceptibility to URT disorders. The Boston terrier, boxer, CKCS, English and French bulldog and pug are brachycephalic breeds and affected to a variable degree by BOAS^5,33–35^. The Norwich terrier, often classified as a mesaticephalic breed, suffers from Norwich Terrier Upper Airway Syndrome (NTUAS), an obstructive disorder that has features similar to, but also distinct from BOAS^11,36^. The Irish wolfhound is susceptible to rhinitis, and respiratory infections^5^. The Chihuahua, CKCS, Pomeranian and Yorkshire terrier are breeds frequently affected with the URT disorder tracheal collapse^5,37–40^.

Although the brachycephalic breeds most often cited in association with URT disorders are the English and French bulldog, the pug and the Boston terrier, our study found that the Japanese chin, Pomeranian, CKCS and boxer also have an increased risk for URT disorders. Among 13 breeds with high RR for URT disorders in this study, eight (Boston terrier, boxer, CKCS, English bulldog, French bulldog, Japanese chin, Pomeranian, pug) are recognised as brachycephalic by most researchers^28,41,42^. However, depending on the definition of brachycephaly, some authors suggest that the Chihuahua may be included in this category or that the Pomeranian be excluded^7,42,43^. There are currently 25 recognized brachycephalic breeds among the 327 breeds in the database^28^. This shows a definite overrepresentation of brachycephalic breeds among breeds most at risk for URT disorders. The brachycephalic conformation has been linked to an increased risk for URT disorders, and specifically BOAS, in the French and English bulldog, the CKCS, Boston terrier and the pug^4–7,42–44^. This study has the advantage of presenting further and strong evidence to this fact, by investigating a large sample population, which is representative of the Swedish dog population^15^. Some brachycephalic breeds have had a recent surge in popularity, which has in turn led to an increased frequency of veterinary care events for health issues commonly affecting these breeds. For example, a recent study found that corrective surgery for BOAS markedly increased in Swedish veterinary clinical practice between the years 2014 and 2016^45^. However, it remains unclear to which extent the brachycephalic conformation contributes to the increased risk for URT disorders found in this study for the Japanese chin, Pomeranian and boxer. Further studies are necessary to investigate if there is a conformational inference to the URT disorders found in these breeds.

Male dogs had a slightly increased risk for URT disorders, which is in accordance with an earlier Swedish study reporting a male predisposition to respiratory disease^1^. Stratified by breed within high-risk breeds, this predisposition was significant only for the pug; male pugs had twice the odds of URT disorders compared to females. This result contrasts findings in a UK-based study in which female pugs were five times more at risk of BOAS compared to males^44^. Possible reasons for this might be an actual difference between pug populations in the two countries or that the recruited population used in the single-centred UK study was not entirely representative of the national pug population (selection bias).

Median age at first URT diagnosis varied with breed, most probably reflecting the different age that various disorders manifest. Laryngeal paralysis for example typically affects older dogs, NTUAS middle-aged to older dogs, whereas BOAS presents at a younger age^11,28,39^. Median age at first diagnosis was significantly lower for the English and French bulldog, pug, boxer and Chihuahua compared to all other breeds with URT diagnosis. A possible explanation for this is that these breeds are affected with congenital and/or developmental disorders, which as a rule present early in life. A sudden surge in breed popularity, as has been noted for the Chihuahua and the French bulldog^14,46–48^, might also lead to a generally younger population of these breeds presented in the database with URT diagnosis, with relatively fewer older dogs registered with an URT diagnosis. In contrast, breeds with constant registration numbers would have a population with a more balanced age distribution. If the bulk of dogs present in the dataset are relatively young, this will bias the age at first URT diagnosis downwards in these breeds. Among 13 high-risk breeds, eight brachycephalic breeds were diagnosed with an URT diagnosis almost one year earlier than five non-brachycephalic breeds. Several factors can contribute to this, for example a predilection of brachycephalic breeds to congenital and/or developmental disorders, or a relatively young population in some of the brachycephalic breeds in the database, as earlier mentioned. However, it is still noteworthy that brachycephalic breeds acquire a URT-related disease burden earlier in life compared to non-brachycephalic breeds, as this disorder may have a negative influence on their life quality for a larger proportion of their lives compared to the non-brachycephalic breeds.

The average lifespan of dogs with URT disorders was 7.81 years in this study. Recent studies based on collection of primary care data published a 12- and 10-year average life expectancy for the general dog population in the UK and Denmark respectively^49–51^. However, life expectancy calculations based on life insurance data, which pertain an inherent upper age limit will probably bias the average lifespan downwards compared with primary care data or questionnaire studies observing dogs throughout their lifetime. Longevity varies with dog breed, and is generally inversely related to breed size, with large-sized and giant breeds usually having a shorter lifespan than medium- and small-sized breed dogs^49–51^. With the exception of the Irish wolfhound, all other high-risk breeds in this study were of medium to small size, and therefore an average lifespan of 10 to 12 years might be expected^49–52^. Notably, French bulldogs with URT disorders had a dramatically shorter lifespan (3.6 years) than the normal life expectancy in this small-sized dog. The average lifespan recently reported for the French and English bulldog and the pug (8.6 years) was approximately 3 years shorter compared to like-sized mesaticephalic and dolichocephalic breeds^4^. The authors suggested that BOAS and other conformational disorders common in these breeds lead to an earlier death^4^. French bulldogs have higher odds for several serious health issues compared to the average dog population^53^. Many of these predispositions have been attributed to their extreme brachycephalic conformation, suggesting that breeding for a less extreme exterior could improve the general health in this breed^53^. The short average lifespan of French bulldogs with URT disorders in our study is probably the result of an exceptionally high disease burden in this breed. However, a relatively young population of French bulldogs in the database (due to a sudden surge in popularity of this breed) could also contribute to a lower age at death. Moreover, attitudes to disease, death, and euthanasia among owners and veterinarians will also affect a dog’s lifespan. These attitudes can vary significantly between countries due to societal differences. The impact of such attitudes on the decision to pursue continued health care or opt for euthanasia can be hard to measure.

Most of the high-risk breeds for URT disorders had increased odds for co-morbidities; thereby appearing to be less healthy than the general dog population. This could reflect an actual higher disease burden in these breeds, as has for example been shown for the French bulldog^53^. The English bulldog and the CKCS are also prone to a high disease load^5^. In Norway recently, a court ruled that breeding English bulldogs and CKCSs contravened the country´s animal welfare act, and imposed a ban on these breeds. However, the court allowed for modified breeding practices, such as crossbreeding, aiming to alleviate these breeds’ significant health problems^54^. Although our study provides some information on the disease burden of high-risk breeds for URT disorders, it was not designed to present comprehensive breed-specific disease predispositions. Studies investigating specific breeds’ disease susceptibilities are relatively scarce in the veterinary literature. Such studies could provide information on the overall health issues of individual breeds, which would be valuable for veterinarians, breeders, prospective dog owners, and policy makers. Economic factors, veterinary care availability and personality traits might have an impact on owners’ decision to seek veterinary care^55^. Affluent owners with proximity to veterinary care and owners with a strong bond to their dogs may be more prone to seek veterinary care, contributing thus to a seemingly bigger disease load in their dogs.

Epidemiologic data can be collected by different methods, each subject to limitations. Questionnaire-based surveys are easy to distribute and may be representative of the entire population, but are subject to potentially low response rates, diagnostic inaccuracy and responder and recall bias. Data from referral hospital records have high diagnostic and demographic accuracy, but introduce a potential selection bias, because more complex clinical cases will be represented in this material. Data from primary care records may be more representative of the population at risk than referral hospital data, but similar to referral hospital data, contain no information on the healthy background population. Large-scale epidemiologic data can be retrieved from insurance databases, which, unlike hospital data, contain information on both cases and the insured healthy background population, from the start until the end of a dog´s insurance period. When a sufficient proportion of a country´s dog population is insured, findings can be generalised to the national dog population. In Sweden, 77% of all dogs were covered by veterinary insurance, with approximately 50% of those enrolled in Agria Djurförsäkring during the year 2012^13,14^. The Agria Djurförsäkring database has been validated against veterinary practice records and found to have excellent agreement (> 94%) for demographic data such as breed and sex, and fair agreement (85%) for diagnostic accuracy and date of birth, thus rending the quality of the database adequate to support ongoing research^56^. Another advantage is that the dataset is large, allowing for significant statistical power, with the exception of rare breeds with a limited number of individuals.

Limitations associated with utilising insurance databases in research have been described in detail^55^ but some that apply to this study must be mentioned. Selection bias introduced by claim excess amounts and condition exclusions from pet insurance cover lead to an unknown number of unregistered cases. A veterinary care event must rend a cost exceeding the deductible amount for the event to be registered in the database, thus a number of cases will go undetected. However, this most probably affects the general IR of URT disorders, and not breed-specific results. During the study observation period, the only URT-related exclusion from insurance coverage was for the surgical treatment of conditions affecting the nostrils, soft palate, trachea and pharynx in the Boston terrier, French and English bulldog and pug. Further, several studies report that owners and veterinarians will often accept clinical signs of BOAS until they are severe, which consequently adds to the number of undetected and undiagnosed cases^9,43,57^. Consequently, the true incidence of URT disorders in these breeds is most probably much higher than reported in this study.

Another limitation is that results from epidemiological studies based on insured populations may not be valid in uninsured animals^55^. In Sweden, although the vast majority (77% percent) of dogs were insured in 2012^14^, there may still be differences between the insured and uninsured dog population; an earlier study found that insurance coverage varied somewhat with breed^58^.

The inclusion of tracheal disorders in this study may be considered controversial, as the trachea is usually classified as part of the lower respiratory tract in the literature. The distinction between upper and lower airway is not clear, and some researchers might even include the larynx in the lower airway. However, many clinicians and part of the veterinary literature consider the cervical trachea and the larynx as part of the upper airway^23,24,59^. As the diagnostic codes in this study were assigned by clinicians, we have chosen to include the trachea in our list of URT diagnoses.

Finally, BF correction was applied to adjust for multiple comparisons, but it should be noted that the likelihood of type II errors increases when BF correction is applied. However, as the sample size in the current study is large, a type II error should be regarded as less probable.

## Conclusions

In conclusion, URT disorders affected almost 5000 dogs in this study population, with an incidence of 50.56 (95 CI; 49.14–52.01) cases per 10,000 DYAR. Our study confirmed previously known breed predispositions for URT disorders, and identified two unreported breeds at risk. Besides BOAS in brachycephalic breeds, a breed predisposition for other URT diagnoses in non brachycephalic breeds were verified. We found a definite overrepresentation of brachycephalic breeds among breeds with high RR for URT disorders. The epidemiological information associated with URT disorders in this study can be of value for veterinarians in clinical- and research settings. In addition, our results provide educational information on breed-related health risks for breeders, dog owners and policy makers.

## Supplementary Information

Supplementary Information.

## Data Availability

The data analysed in the current study are not publicly available due to a non-disclosure agreement with Agria Djurförsäkring.

## Abbreviations

BF: Bonferroni
BOAS: Brachycephalic obstructive airway syndrome
CI: Confidence interval
CKCS: Cavalier King Charles Spaniel
DYAR: Dog years at risk
IQR: Interquartile range
IR: Incidence rate
NTUAS: Norwich terrier upper airway syndrome
RR: Relative risk
URT: Upper respiratory tract
