# Supplementary material for: The epidemiology of upper respiratory tract disorders in a population of insured Swedish dogs (2011–2014), and its association to brachycephaly
Source: Sci Rep. 2023 May 30;13:8765. doi: 10.1038/s41598-023-35466-0 (PMC10229607; doi:10.1038/s41598-023-35466-0)
Supplement: Supplementary file 1 — Supplementary Information. [file 41598_2023_35466_MOESM1_ESM.docx]

**The epidemiology of upper respiratory tract disorders in a population of insured Swedish dogs and its association to brachycephaly.**

Dimopoulou M^1*^, Engdahl K^1^, Ladlow J^2^, Andersson G^3^, Hedhammar Å^1^ Skiöldebrand E^4^ & Ljungvall I^1^.

Affiliations:

^1^Department of Clinical Sciences, Swedish University of Agricultural Sciences, Uppsala, Sweden.

^2^Department of Veterinary Medicine, University of Cambridge, UK.

^3^Department of Animal Breeding and Genetics, Swedish University of Agricultural Sciences, Uppsala, Sweden.

^4^Department of Biomedical Science and Veterinary Public Health, Swedish University of Agricultural Sciences, Uppsala, Sweden.

^*^Corresponding Author; e-mail: Maria.Dimopoulou@uds.slu.se

**Supplementary table 1**. Age at termination of life insurance by breed for dogs insured in Agria Djurförsäkring (2011-2016)

| **Age group 12** | Bichon Havanais, Border Terrier, Cairn Terrier, Chihuahua, Chinese Crested, Miniature Schnauzer, Finnish Lapphund, Finnish Spitz, Fox Terrier, Islandic Sheepdog, Jack Russel Terrier, Lhasa Apso, Poodle (toy, miniature, medium), Münsterländer, Norrbottenspets, Norwegian Buhund, Papillon, Phalene, Schnauzer, Shih Tzu, Soft Coated Wheaten Terrier, Tibetan Spaniel, Tibetan Terrier, Västgötaspets, Welsh Springer Spaniel, West Highland White Terrier, Whippet |
| --- | --- |
| **Age group 10** | All other breeds |
| **Age group 8** | Berner Sennen, Grand Danois, Irish Wolfhound, Leonberger, Newfoundland, Pyrenean Mountain Dog, Neapolitan Mastiff, St. Bernard |

**Supplementary Table 2**. List of upper respiratory tract diagnostic codes included in the analyses.

| RA0 | Normal variation, symptom with no confirmed etiology, upper airways |
| --- | --- |
| RA01 | Symptom of disease with no confirmed etiology, upper airways |
| RA012 | Stenosis / noise from upper airways during exercise |
| RA013 | Cough from upper airways |
| RA014 | Dyspnea |
| RA015 | Reversed sneezing |
| RA1 | Congenital, developmental, growth condition, upper airways |
| RA10 | Congenital deformity, upper airways |
| RA102 | Stenosis, dysfunction of the nares |
| RA11 | Developmental abnormality, growth anomaly, upper airways |
| RA111 | Palatum molle elongatum |
| RA112 | Brachiocephalic obstruction |
| RA113 | Laryngeal collapse |
| RA2 | Metabolic, nutritional, degenerative, dystrofic conditions, upper airways |
| RA20 | Metabolic conditions, upper airways |
| RA22 | Degenerative/dystrofic conditions, upper airways |
| RA221 | Laryngeal paralysis |
| RA3 | Circulatory conditions, upper airways |
| RA30 | Bleeding, upper airways |
| RA31 | Oedema, upper airways |
| RA311 | Laryngeal oedema |
| RA312 | Pharyngeal oedema |
| RA313 | Glottic oedema |
| RA32 | Infarcts, upper airways |
| RA33 | Hygromas, upper airways |
| RA4 | Infectious, inflammatory conditions, upper airways |
| RA41 | Acute inflammatory conditions, upper airways |
| RA411 | Acute serous / serofibrinous inflammation, upper airways |
| RA412 | Acute purulent inflammation, upper airways |
| RA413 | Specific acute inflammatory conditions, upper airways |
| RA419 | Other acute inflammatory conditions, upper airways |
| RA4192 | Acute pharyngitis |
| RA4193 | Acute laryngitis |
| RA42 | Chronic inflammatory conditions, upper airways |
| RA421 | Chronic serous inflammation, upper airways |
| RA429 | Other chronic inflammatory conditions, upper airways |
| RA4194 | Acute epiglottitis |
| RA4292 | Chronic pharyngitis |
| RA4293 | Chronic laryngitis |
| RA4294 | Chronic epiglottitis |
| RA4299 | Other chronic upper airway infections |
| RA711 | Roaring |
| RA73 | Position changes, upper airways |
| RA731 | Dislocation of the soft palate |
| RA732 | Entrapment |
| RA733 | Epiglottic retroversion |
| RA9 | Idiopathic, unspecified, multifactorial conditions, upper airways |
| RB0 | Normal variation, symptom with no confirmed reason, lower airways |
| RB01 | Symptom of condition with no confirmed reason, lower airways |
| RB012 | Dyspnoea from upper, lower airways |
| RB013 | Apnea |
| RB014 | Forced breathing |
| RB1 | Congenital, developmental, growth condition, trachea, lower airways |
| RB10 | Congenital condition, trachea, lower airways |
| RB101 | Collapsed trachea |
| RB102 | Tracheal hypoplasia |
| RB11 | Developmental, growth condition, trachea, lower airways |
| RB4291 | Chronic tracheitis |
